# Bridging biology and therapy: translational advances of extracellular vesicles in veterinary clinical practice

**DOI:** 10.1007/s11259-025-10917-3

**Published:** 2025-11-17

**Authors:** Walaa A. Gad, Sally Ibrahim, Hiam Nagdy, Bassma S. M. Elsawy, Dina Aboelsoued, Hoda S. M. Abdel-Ghany, Ahmed A. A. Abdel-Wareth, Khaled A. Abd El-Razik, Karima Gh. M. Mahmoud, Walid T. M. Soliman, Mohamed O. Taqi

**Affiliations:** 1 Department of Animal Reproduction & Artificial Insemination, Veterinary Research Institute, National Research Centre, Giza, Egypt; 2 Department of Parasitology and Animal Diseases, Veterinary Research Institute, National Research Centre, Giza, Egypt; 3 Department of Animal and Poultry Production, Faculty of Agriculture, South Valley University, Qena, 83523 Egypt; 4 Cooperative Agricultural Research Center, College of Agriculture, Food and Natural Resources, Prairie View A&M University, Prairie View, TX 77446 USA; 5 Department of Development of Animal Wealth, Faculty of Veterinary Medicine, Egyptian Chinese University, Cairo, Egypt; 6 Central Laboratory for Agricultural Climate, Agricultural Research Center, Ministry of Agriculture and Land Reclamation, Dokki, Giza Egypt

**Keywords:** Extracellular vesicles, Infectious diseases, Drug delivery, EV-based vaccines, Therapeutic applications, Reproductive disorders

## Abstract

Extracellular vesicles (EVs) are small membrane-bound particles released by numerous cell types and are gaining popularity in veterinary medicine due to their extensive biological activities and therapeutic potential. This review summarizes the classification, biogenesis, and molecular cargo of various types of EVs, such as exosomes, microvesicles, and apoptotic bodies, as well as their emerging roles in cellular communication, diagnostics, and therapeutics across a wide range of veterinary applications. Beyond mesenchymal stem cell (MSC)-derived EVs, EVs from immune cells, pathogens, and body fluids show great promise for tissue healing, immunological regulation, infectious disease management, drug delivery systems, vaccine development, and reproductive health. We critically evaluate recent advancements, limitations, and future possibilities in using EVs to improve diagnosis and treatment results in veterinary species. The review’s goal is to provide a comprehensive picture of the rapidly increasing EV landscape and to make it easier to incorporate EV-based technology into clinical veterinary practice.

## Introduction

**Fig. 1:**
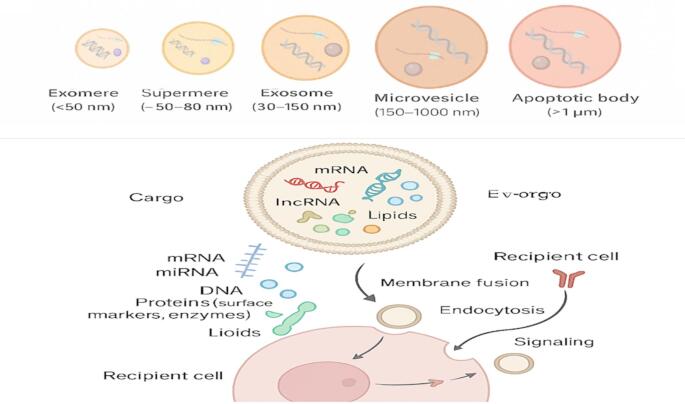
Overview of extracellular vesicle subtypes, cargo, and intercellular communication mechanisms

Extracellular vesicles (EVs) are lipid bilayer-bound particles ranging from 30 to 5000 nm, released by virtually all cell types, ranging from prokaryotes to higher eukaryotes and present in numerous biological fluids including blood, urine, and saliva (Liangsupree et al. 2021). EVs are broadly classified into three main types according to their biogenesis, size, and function: exosomes (30–150 nm), microvesicles (100–1000 nm), and apoptotic bodies (> 1000 nm) (Fig. 1) (Zaborowski et al. 2015).

Exosomes are released into the extracellular environment when MVBs merge with the plasma membrane (Xu et al. 2023). These vesicles contain various bioactive components, such as lipids, proteins, DNA, and RNA, which mirror the molecular profile of their originating cells. Exosomes involve various biological processes such as antigen presentation, immune modulation, and tissue repair. Due to their size, stability, and cargo-carrying capacity, exosomes are increasingly recognized as promising tools for therapeutic delivery (Liu 2024) Apoptotic bodies are generated during apoptosis, a programmed cell death process, and contain fragments of the dying cell, including cellular organelles, DNA, and other components.

Apoptotic bodies serve as a mechanism for removing dead or dying cells and may influence immune responses by presenting cellular debris to immune cells, such as macrophages, which help clear the remnants. While apoptotic bodies have less therapeutic potential than exosomes or microvesicles due to their heterogeneous cargo and larger size, they still play an essential role in maintaining homeostasis and modulating immune responses in the body (Liu et al. 2022).

Microvesicles are directly released from the membrane of plasma through outward budding (Clancy et al. 2021). The cytoskeleton regulates this process and involves the shedding of portions of the membrane, which contain lipids, proteins, and RNA that are characteristic of the parent cell. Microvesicles participate in cellular communication, inflammation, coagulation, and tumor progression. Their larger size compared to exosomes makes them particularly suitable for certain therapeutic applications, such as tissue regeneration and immune modulation (Lanci et al. 2024). In addition to the classical EV subtypes, recent research has identified novel classes, including exomeres (< 50 nm), supermeres, and large oncosomes (> 1 μm), each with unique biogenesis pathways and biological roles (Zhang et al., 2022).

Each type of EV contains a distinct set of bioactive molecules that reflect the state of physiology and health of their parent cells, making them valuable in understanding disease mechanisms and developing targeted therapies evasion (Xu et al. 2023). For example, exosomes derived from mesenchymal stem cells (MSCs) have been shown to carry regenerative factors that promote tissue healing and immune modulation, while microvesicles have been linked to tumor progression and immune evasion (Xu et al. 2023).

The characterization of these vesicles, including their size, cargo, and origin, is essential for harnessing their therapeutic potential and understanding their role in various biological processes (Lanci et al. 2024). As their ability to transport diverse cargo and modulate cellular processes, extracellular vesicles are being increasingly investigated as delivery vehicles for therapeutics. Their biocompatibility, low immunogenicity, and natural ability to target specific cells or tissues make them promising candidates for drug delivery and gene therapy applications (Lanci et al. 2024). Several approaches have been developed to isolate EVs, each of which has specific advantages and drawbacks according to yield and purity (Ma et al. 2025).

Extracellular vesicles are isolated from biological fluids, cell culture and other sources (Carnino et al. 2019). Sample yield and purity are significantly influenced by the technique applied to isolate EVs. Usually, freshly collected and processed materials generate more vesicles (Ma et al. 2025). According to Ramirez et al. (2018), impurities that may coseparate with EVs, such as cellular debris, in addition to interfering with components such as lipoproteins, protein complexes, and aggregates, make EV isolation more difficult than just their nanosize (Chiaradia et al. 2021).

They are shed from most cells and carry proteins, lipids, and adenosine‒triphosphate ions such as calcium and nucleic acids (Gratpain et al.^2021^). The cargos of EVs perform various biological functions that are essential for mother–fetus communication, immune surveillance, inflammation and oxidative stress (Cho et al. 2020).

They also regulate a broad range of pathological processes in neurological disorders (Gratpain et al. 2021) and cardiovascular diseases (Han et al. 2022a) while playing crucial roles in the formation of the premetastatic niche (Becker et al. 2016). Extracellular vesicles have attracted significant attention due to their ability to facilitate communication between cells by delivering bioactive molecules, including lipids, proteins, and nucleic acids (Liu 2024). These vesicles are now recognized not only for their physiological roles but also for their therapeutic potential in both human and veterinary medicine, particularly for regenerative therapies in animals (Xu et al. 2023).

Recent veterinary research emphasizes the role of extracellular vesicles (EVs) in numerous species, including dogs and horses (Lanci et al. 2024). Studies indicate EVs’ critical involvement in cancer biology, including tumor development and metastasis, as well as their ability to influence immune responses (Kuang et al. 2025). For example, canine tumor-derived EVs have been demonstrated to alter immune cell activity, contributing to immune evasion, whereas equine EVs have a role in inflammatory modulation during musculoskeletal injuries (Kang et al. 2025). These findings highlight the translational potential of EVs as biomarkers and therapeutic agents in veterinary clinical practice.

Extracellular vesicles are rapidly being recognized as important modulators of immune responses in veterinary species. For example, platelet-derived vesicles in dogs have been shown to be ingested by T lymphocytes, regulating their activation and cytokine production (Kang et al. 2025). This emphasizes the importance of EVs as active immunological regulators, which has implications for both infectious and autoimmune disorders in animals.

The significance of EVs in promoting intercellular communication is retained throughout a wide range of animals, including mammals and plants. Cross-species studies show that EVs can mediate signaling both within and between organisms, underscoring their fundamental biological importance and potential for novel therapeutic applications (Liu 2024).

Extracellular vesicles (EVs) comprise diverse nanoscale particles released by cells, classified by size, biogenesis, and function. Classical subtypes include exosomes (30–150 nm), microvesicles or ectosomes (150–1000 nm), and apoptotic bodies (> 1000 nm). Emerging classes such as exomeres (< 50 nm), supermeres, and large oncosomes (> 1 μm) are also represented. EV cargo includes proteins, lipids, nucleic acids, and metabolites that mediate physiological and pathological signaling. EVs interact with recipient cells via ligand-receptor binding, membrane fusion, and endocytosis to transfer molecular signals and regulate cellular functions.

The functions of extracellular vesicles (EVs) in intercellular communication and other physiological processes are facilitated by a number of significant biological characteristics.

## EV main biological characteristics

1. **Composition specificity**: EVs have a distinct set of proteins, lipids, and nucleic acids (such as RNA) that represent their cell of origin and can affect their function and interaction with destination cells. EVs reflect the characteristics of their source cells. Proteins such as CD63 and HSPA8 are often found in EVs and can be used to differentiate diseases such as cancer, atherosclerosis, and osteoporosis (Oggero et al. 2021). EVs can also carry RNA, miRNAs, and DNA, which serve as valuable noninvasive biomarkers for various diseases. The specificity of EV composition enables their use in diagnostic and therapeutic contexts, as their cargo mirrors physiological and pathological cellular states.
2. **Target specificity**: The ability of EVs to target particular cells or tissues allows for precise cargo delivery and communication. Surface proteins frequently mediate this targeting by interacting with recipient cell receptors. On the basis of surface proteins such as integrins, which determine which organs and tissues EVs target, EVs can attach to particular cells (Wortzel et al. 2019). These are helpful in studying disease mechanisms and treating diseases such as cancer because of their specific targeting ability.
3. **Circulatory Stability**: EVs are stable in circulation, which allows them to survive in the bloodstream and reach distant sites in the body. Their lipid bilayer protects their contents from degradation by enzymes (Akers et al. 2016). The biological activity of EVs is influenced mainly by their integrity; if their integrity is compromised, they lose their biological effects. EVs are resilient and can circulate in the bloodstream for extended periods, which is advantageous for drug delivery. Their ability to resist degradation and their potential for surface modification improve their stability and effectiveness in therapy. Consequently, EVs can be chemically modified to lower clearance rates and increase circulation stability. The circulation stability and half-life of biomarkers were further extended by their content and surface modification, even while EVs shield their contents from nuclease and protease degradation. Comprehensive knowledge of the proteins and particular lipids on the surface of EVs is needed.
4. **Active penetration of biological barriers**
  EVs can effectively navigate biological barriers, such as the blood‒brain barrier (BBB) (Chen et al. 2022), making them valuable for treating CNS disorders. They have been shown to effectively deliver drugs and genetic material to targeted areas within the body. A major obstacle for therapeutic agents, showing significant potential for treating cerebrovascular and neurodegenerative diseases (Xu et al. 2023). These properties make EVs a focus of research because of their potential applications in diagnostics, therapeutics, and as drug delivery vehicles in clinical settings.
  1. **Drug delivery vehicles and anticancer vaccines**: EVs, especially those derived from mesenchymal stem cells (MSCs), offer advantages over synthetic drug delivery systems, including their lower immunogenicity and cytotoxicity and better bioavailability and biocompatibility (Tang et al. 2021) than other synthetic drug delivery systems, such as liposomes (Palazzolo et al. 2018). They are also being explored as vehicles for anticancer drugs and as components of immunotherapies, such as vaccines.
  2. **Oxidative Phosphorylation**: EVs, especially those derived from MSCs, carry out oxidative phosphorylation, generating ATP to sustain cell energy and function, which can aid in the healing of damaged tissues (Panfoli et al. 2016).
  3. **Enzyme activity**: Some of the enzymes found in EVs, such as proteases, are involved in immune response modulation, extracellular matrix remodeling, and metabolic regulation. By activating these enzymes under pathological circumstances, the functional potential of EVs is further increased.
  4. **In summary**, EVs are lipid bilayer particles secreted by virtually all cell types, carrying molecular cargo that reflects their origin and determines their function. Their surface proteins direct targeted delivery to specific cells, while their stability in circulation allows them to act as durable messengers and drug carriers. Notably, EVs can cross biological barriers such as the blood–brain barrier, enhancing their therapeutic reach. Derived EVs, particularly from MSCs, exhibit regenerative, immunomodulatory, and enzymatic activities, making them valuable tools in diagnostics, drug delivery, and immunotherapy.

## Processes governing EV formation and release

EXOs originate from the endosomal pathway, specifically from multivesicular bodies (MVBs). These MVBs contain small vesicles created by inward budding of the endosomal limiting membrane, which subsequently fuse with the plasma membrane to release intraluminal vesicles as exosomes (Xu et al. 2023). Once formed, MVBs merge with the cellular membrane, where they secrete their vesicular contents outside of the cell as EXOs (Xu et al. 2023).

This process of fusion is mediated by key membrane proteins, such as RAB35 and RAB11, which facilitate the release of EXOs rich in flotillin (Han et al. 2022a). The endosomal sorting complex required for the transport machinery, which is central to biogenesis, is essential for MVB formation and sorting (Van Niel et al. 2018). The ESCRT machinery comprises several components, including ESCRT-0, ESCRT-1, and ESCRT-3, which control cargo clustering, budding, and vesicle separation (Krylova and Feng 2023). Indeed, the knockout of specific ESCRT proteins such as TSG101 and HRS results in decreased EXO secretion, demonstrating the importance of this machinery for EXO release in many cellular types (Han et al. 2022b).

In addition to the ESCRT machinery, RAB family proteins, such as RAB11, RAB27A, RAB27B, and RAB35, play crucial roles in facilitating the movement and fusion of MVBs with the cellular membrane, thereby regulating EXO release (Krylova and Feng 2023).

These proteins are directly involved in transporting late endosomes to the cellular membrane, orchestrating the secretory pathway, and facilitating the recycling of membrane components (Krylova and Feng 2023). EVs, particularly exosomes, must connect to target cells via specific mechanisms, such as ligand‒receptor interactions, phagocytosis, endocytosis, and micropinocytosis, to deliver their cargo effectively (Montecalvo et al. 2012).

**Fig. 2:**
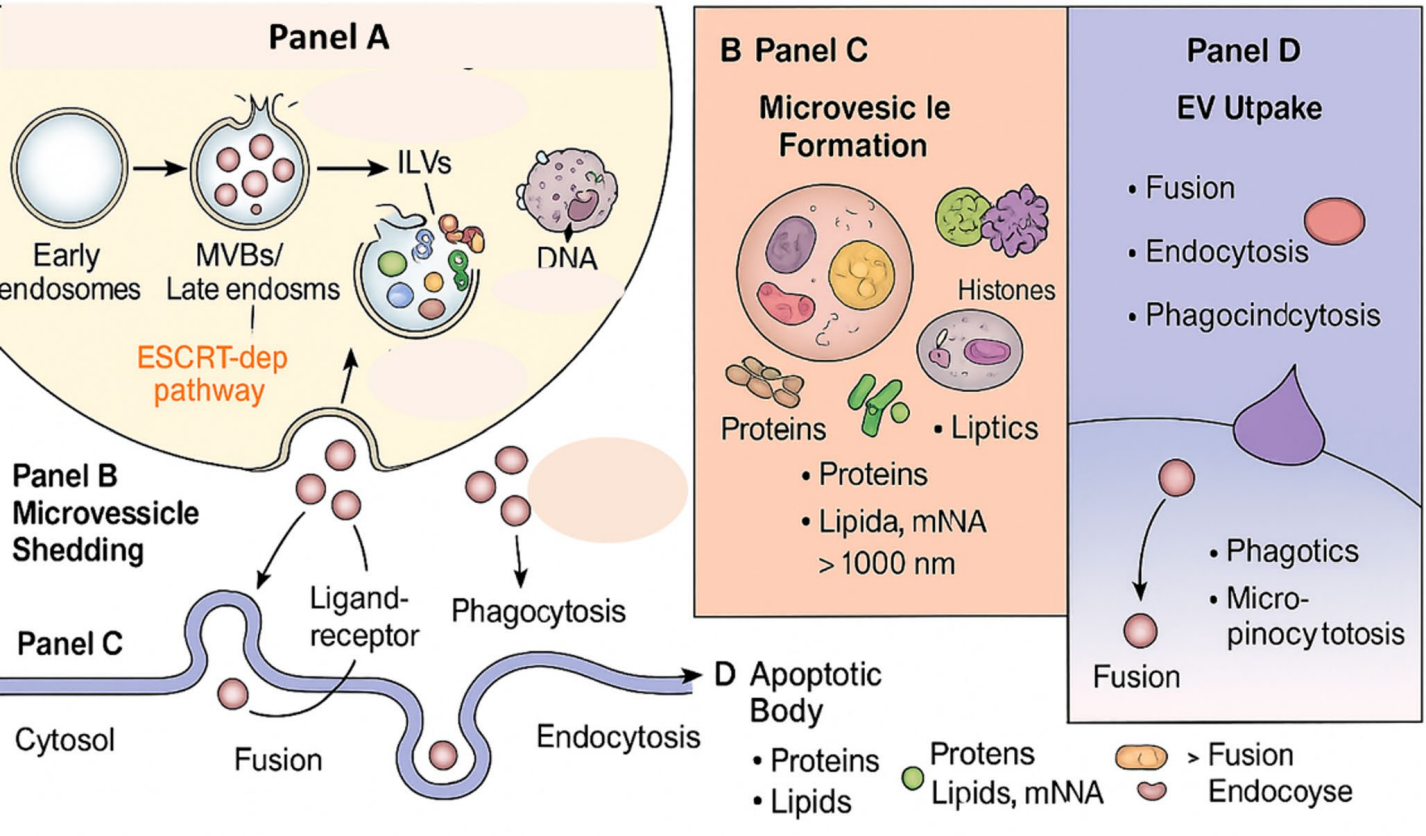
Biogenesis and release mechanisms of major extracellular vesicle (EV) subtypes. Panel A Exosome biogenesis starts with the development of intraluminal vesicles (ILVs) within multivesicular bodies (MVBs), which are generated from early endosomes. This process involves the endosomal sorting complex needed for transport (ESCRT) machinery, as well as auxiliary proteins such tetraspanins and RAB GTPases. MVBs either fuse with lysosomes for destruction or merge with the plasma membrane, releasing exosomes (30–150 nm) into the extracellular environment. Panel B Microvesicle shedding (also known as ectosome production) takes place via direct outward budding of the plasma membrane, which involves cytoskeletal rearrangement (actin remodeling) and phospholipid redistribution mediated by enzymes including floppase and scramblase. Released microvesicles (100–1000 nm) transport proteins, lipids, and nucleic acids. Panel C Apoptotic bodies are formed when cells disassemble during apoptosis, resulting in enormous vesicles (> 1000 nm) that include cellular organelles, nuclear fragments, and numerous biomolecules such as DNA, histones, lipids, and proteins. These vesicles are removed via phagocytic absorption by adjacent or immune cells. Panel D EV absorption by recipient cells happens by a variety of processes, including membrane fusion, ligand-receptor interaction, phagocytosis, micropinocytosis, and endocytosis. This makes it easier to deliver functional cargo including RNAs, proteins, and lipids into recipient cells, hence improving intercellular communication

The surface of EXOs is equipped with adhesion molecules, such as the integrins α6β1 and α6β4, which facilitate cell-specific targeting, particularly in malignancies such as liver and lung cancer (Han et al. 2022b). Furthermore, the acidic environment of the recipient cells enhances the fusion and integrity of EXOs, promoting their interaction with their targets (Han et al. 2022b). The increased understanding of these mechanisms underscores the vital role of EVs in cellular communication, positioning them as promising tools for disease diagnosis and therapy (Fig. 2).

## Methods for extracellular vesicle isolation

### Differential centrifugation

Differential centrifugation is a sequential centrifugation technique involving multiple spins at increasing centrifugal forces (e.g., 300 x g, 2,000 x g, 10,000 x g) to remove cells, cell debris, and larger vesicles (Revenfeld et al. 2014). This method separates particles based on size and density differences, with larger particles sedimenting at lower speeds and smaller particles pelleting at higher speeds (Gardiner et al. 2016). The main advantages of this technique are the capacity to address vast amounts of solutions and separate large quantities of EVs at one time, in addition to the low processing cost, and the lack of necessity for extra chemicals (Konoshenko et al. 2018). The requirements for ultracentrifugation tools and the difficulty of stepwise procedures are considered drawbacks of this method (Gardiner et al. 2016). The time taken by differential centrifugation might range from 140 to 600 min, furthermore limitations include potential co-isolation of impurities and partial EV loss (Lucchetti et al. 2019).

### Ultracentrifugation

Ultracentrifugation refers to centrifugation at very high speeds, typically above 100,000 x g, used to pellet small EVs such as exosomes after removal of larger particles. This method leverages size and density for EV sedimentation but can expose vesicles to high shear stress and may induce vesicle aggregation or damage. It remains the most commonly used method for EV concentration but requires specialized equipment. Typical centrifugation times range from 1 to 4 h depending on rotor type and settings. Regarding Density gradient ultracentrifugation, Extracellular vesicles are separated into distinct layers via density gradient centrifugation, which is based on the density of sucrose, iohexol, and iodixanol in solutions (Cvjetkovic et al. 2014). This technique can effectively separate subcellular components (peroxisomes, mitochondria, endosomes) into specific layers (Konoshenko et al. 2018). Most density gradient approaches are used to separate EVs in which the centrifugation technique has already led to partial isolation. The advantages of this technique include the production of preparations with increased purity and yield and a lack of extra chemicals (Duong et al. 2019). Complications, the need for ultracentrifugation tools, the labor-intensive nature of the technique and sample loss during separation are among the drawbacks of this technique (Konoshenko et al. 2018). Additionally, this approach takes much time, as it takes between 250 min and 2 days to complete (Théry et al. 2018). Crucially, EVs obtained by ultracentrifugation have also been reported to exhibit reduced functioning (Mol et al. 2017). or agglomerate (Linares et al. 2015). This could be related to the harmful stresses applied to the vesicles during high-speed centrifugation.

### Size-based technique

#### Ultrafiltration (UF)

This technique, also known as microfiltration, is a popular technique for isolating EVs on the basis of size via porous membranes (pore sizes of 0.1, 0.22, or 0.45 μm), which filter EVs in suspension by allowing the passage of smaller particles and preventing the passage of large particles (Shirejini and Inci 2022). Typically, this process is carried out in sequential steps to separate EVs of the required size (Lucchetti et al. 2019). This approach is simple, inexpensive, and suitable for tiny and large sample sizes. Ultrafiltration produces EVs that are free-standing single particles, not aggregates, which is advantageous for examination later. Furthermore, since the vesicles are not exposed to the equivalent forces and pressures that ultracentrifugation needs, ultrafiltration dramatically lowers the chance of EV rupture; this probably explains why ultracentrifugation does not recover as many EVs as ultrafiltration does (Yu et al. 2018). Although EVs generated via ultrafiltration are often contaminated with molecules of the same diameter, this method has lost some of its suitability for proteome investigations if utilized alone (Inal et al. 2013). It is best to combine ultrafiltration and ultracentrifugation to prevent this (Parimon et al. 2018).

#### Size-exclusion chromatography

In this technique, biomolecules are isolated via porous beads according to their hydrodynamic radius (Monguió-Tortajada et al. 2019). Filtration is performed via a column of porous beads whose radii are smaller than those of the relevant EVs (Yamamoto et al. 1970). SEC has several noteworthy advantages, including purity, maintenance of vesicle integrity and prevention of EV clustering. This technique is also quick, easy to use and requires one minute for each milliliter of solution. Although SEC is quick and easy while maintaining EV integrity, there is a considerable risk of contamination from particles of the same size as lipoproteins (Guan et al. 2020). The pressure applied to EVs causes sheering, and larger particles might clog the membrane, which represents the drawback of this technique. Moreover, special tools and columns, sample volume restrictions, and technique complexity are needed (Gámez-Valero et al. 2016).

### Immunoaffinity capture-based isolation

To isolate EVs, the immunoaffinity-based microfluidic method relies on the antigen‒antibody response. Affinity-based techniques involve extremely identifiable interactions among receptors located on EV membranes and their analogous ligands, such as antibodies (Liangsupree et al. 2021). Immunoaffinity capture, which uses antibodies against EV surface proteins, is the most widely used affinity-based technique for EV separation. This approach is especially valuable when high specificity is required, such as isolating EVs from complex biological fluids like blood, where EVs derive from multiple tissue sources, which is considered a primary advantage. Moreover, it saves time, is simple, has high purity, is compatible with routine laboratory equipment, and allows precise interactions to target EV-specific markers. Some limitations of this technique are the need for expensive reagents, its limited yield, as it can only be used with a low volume of samples, its inability to be used for large-scale production, and its requirement for prepurification procedures (Komuro et al. 2022).

### Precipitation techniques

This method depends on the use of extremely hydrophilic polymers to precipitate EVs via organic solvents such as polyethylene glycol (PEG), sodium acetate, or protamine (Gallart-Palau et al. 2015). This method is easy to conduct, requires no complicated equipment, saves time, and can process large volumes of samples. On the other hand, the total exosome isolation reagent and the protein organic solvent precipitation technique are expected to be the most efficient and rapid ways to isolate EVs (Gallart-Palau et al. 2015).

When an organic solvent is used, this technique eliminates unwanted soluble protein from biological fluid and results in double-membrane vesicles in suspension, which are consequently separated by centrifugation (Gallart-Palau et al. 2015). Further evidence has shown that precipitating EVs in culture supernatants via sodium acetate is an easy and affordable technique. EVs purified by ultracentrifugation are said to be identical to those isolated via this “salting out” method (Brownlee et al. 2014).

There is a significant chance that non-EV materials such as protein aggregates, polymeric materials, other vesicles, or lipoparticles coprecipitate. Commercial kits that depend on preisolation and postisolation steps are currently available to reduce the risk of contamination with subcellular particles. However, contradictory research indicates that the functional characteristics of isolated EV samples are not adversely affected by copurified components (Ludwig et al. 2018). Some researchers propose that precipitation techniques could negatively impact EV biological functions (Baranyai et al. 2015), particularly given the possible cytotoxic impact and decreased viability noted in certain cell lines after exposure to vesicles isolated through PEG precipitation (Gámez-Valero et al. 2016).

### Microfluidic technologies

Microfluidic-based methods are considered recent techniques for separating EVs from biological fluid. Three groups of microfluidic techniques for EV isolation have been identified: size-based, immunoaffinity-based, and dynamic. Furthermore, size-based devices for EV separation include nanofilters, nanoarrays, and nanoporous membranes (Iliescu et al. 2019). Rapid processing, cost effectiveness, small sample quantities, and high purity and yield are considered advantages of this method (Salafi et al. 2017). The drawbacks of this technique are that it is expensive and that the devices required are highly complex (Konoshenko et al. 2018).

### Limitations of EV isolation methods in regarding subtypes

It should be noted that most conventional EV isolation methods such as ultracentrifugation, density gradient centrifugation, and size-exclusion chromatography do not achieve complete discrimination between exosomes and microvesicles due to the significant overlap in their physical properties (size, density) and the lack of universally accepted, exclusive markers for each subtype (Kong et al. 2025). As a result, preparations obtained by these methods may contain mixed populations of EVs, primarily in the exosome and microvesicle size range. While immunoaffinity-based approaches allow for more selective isolation by targeting membrane surface proteins, these are generally not feasible for large-scale isolation or comprehensive recovery of all EV subtypes. Therefore, downstream characterization steps are recommended to assess the purity and identity of the isolated vesicle populations. This represents a general limitation in EV research and should be considered when interpreting the data (Kong et al. 2025).

Extracellular vesicle preparations were quantified using nanoparticle tracking analysis (NTA) to determine particle concentration. For all downstream applications and comparative experimental groups, EV input was normalized based on particle number to ensure consistent vesicle dosing. Total protein concentration was also measured using a bicinchoninic acid (BCA) assay as a quality control measure. This normalization strategy allowed for the reliable comparison of EV effects between groups. (Peruzzi et al. 2024)

The purity and identity of isolated EV preparations were assessed using multiple complementary techniques. Transmission electron microscopy (TEM) was performed to confirm the presence of vesicular structures with characteristic morphology and size. Western blot analysis was conducted to detect canonical EV markers such as CD63 and TSG101, and to ensure the absence of contaminating cellular proteins (Caglar 2025). These characterization steps are essential to validate the isolated EV populations and support reproducibility and interpretability of functional studies.

### Extracellular vesicles: orchestrating intercellular communication with diverse cargo

EVs serve as significant facilitators of intercellular communication, transferring diverse biomolecules, including RNAs, DNAs, proteins, metabolites, and lipids, as illustrated in Fig. 1. Their selective cargo packaging allows them to influence target cells and regulate various cellular functions. EVs exhibit distinct profiles of proteins and lipids that differ according to their origin, stimuli, and isolation methods. Their protein cargo comprises diverse molecules, including heat shock proteins, cytoskeletal elements, and vesicle trafficking molecules, while their lipid content is enriched with sphingomyelin, cholesterol, and ceramide, among others (Duval et al. 2024). These molecular components allow EVs to function both physiologically and pathologically contexts, highlighting their versatility as biological messengers (Duval et al. 2024).

EVs carry a complementary and dynamic repertoire of nucleic acids, including messenger RNAs (mRNAs), long non-coding RNAs (lncRNAs), and microRNAs (miRNAs), which collectively contribute to post-transcriptional gene regulation in recipient cells. This complex nucleic acid cargo supports EVs’ multifaceted roles in modulating processes such as development, immune responses, and disease progression (Marocco 2025).

The identification of EV-associated RNAs, particularly microRNAs (Da Silveira et al. 2012), has shed light on their critical role in specific biological functions, such as embryo‒maternal communication during implantation (Ng et al. 2013). Selective miRNA cargo within EVs, such as miR-30d, actively modulates recipient cell physiology and has been implicated in gene regulation crucial for implantation success (Vilella et al. 2015). Importantly, the molecular composition of EV cargo is highly context-dependent, adapting dynamically in response to physiological states, environmental stimuli, and pathological conditions. This adaptability enables EVs to act as specialized conveyors of cellular status, mediating functions ranging from tissue regeneration and immune modulation to facilitation of tumor progression and metastasis (Alves et al. 2021).

EVs possess a remarkably dynamic composition that fluctuates in response to the health or disease conditions of their parent cells. This adaptability empowers EVs to act as specialized intermediaries in intercellular communication, playing a vital role in a wide range of biological functions, including cellular proliferation, differentiation, gametogenesis, embryogenesis, and stress response. In physiological contexts, EVs contribute to the governance of normal cellular functions, such as facilitating embryogenesis and orchestrating immune responses (Fazeli and Godakumara 2024). In contrast, during pathological states, EVs can exhibit modified cargo profiles that reflect disease-specific signatures. For example, cancer-derived EVs often carry oncogenic molecules that promote tumor progression, invasiveness, and immune modulation (Suchorska and Lach 2016). Similarly, stress conditions such as hypoxia or inflammation can lead to the packing of EVs with stress-related proteins and microRNAs, aiding in cellular adaptation and survival (Gebremedhn et al. 2020).

This situational plasticity of EV composition underscores their dual role in maintaining cellular homeostasis under healthy conditions and contributing to disease pathogenesis when homeostasis is disrupted (Thiyagarajah 2025). The unique molecular composition of these entities provides critical insights into cellular well-being and has significant potential for advancing diagnostic and therapeutic approaches across diverse medical disciplines. In addition to their involvement in immune-mediated pathogenesis, their pathophysiological role in the development and progression of different diseases, including infectious diseases, and their role as potential therapeutic targets (Gurjar et al. 2025).

### Role of EVs in the most prevalent infectious disease (mastitis)

Extracellular vesicles contribute substantially to mastitis by mediating intercellular signaling and modulating immune responses. During mastitis, infection-induced changes in the bioactive molecules of EV cargo, such as RNAs and proteins, reflect disease-specific signatures, making them valuable biomarkers for early diagnosis. These vesicles not only offer diagnostic insights but also present a promising pathway for delivering drugs to specific targets, enabling the transport of therapeutic agents directly to affected tissues. By leveraging their natural ability to encapsulate and deliver bioactive molecules, EVs hold significant potential for improving mastitis management and enhancing treatment efficacy (Sun et al. 2021).

Extracellular vesicles have attracted significant attention because of their ability to modify the functional state of target cells in addition to their potential use as diagnostic and therapeutic tools. This study provides a complete overview of EV formation, secretion mechanisms, and their role in cellular communication. Furthermore, these findings shed light on their specific involvement in bovine mastitis under clinical and subclinical conditions, highlighting infection-induced changes in EV cargo, their use as biomarkers, and their emerging role in targeted drug delivery (Sun et al. 2021).

EVs, particularly exosomes, are increasingly recognized as essential tools for understanding and managing bovine mastitis. They serve as both mediators of the immune response, inflammation regulation, and tissue repair, as well as promising diagnostic and monitoring tools for mastitis in its clinical and subclinical phases. This dual functionality stems from the fact that the contents of EVs mirror the cellular states of their origins.

Extracellular vesicles involved in mastitis are derived from multiple biological fluids, primarily milk, peripheral blood, and urine, each offering unique insights into disease pathophysiology and potential diagnostic markers.

Milk-derived EVs (mEVs) have been extensively studied given their direct proximity to the infected mammary gland, making them highly relevant for understanding local immune modulation. Proteomic analyses of mEVs have revealed the presence of host defense proteins, including acute-phase proteins and components of neutrophil extracellular traps (NETs), which play key roles in bacterial neutralization and activation of immune pathways at the site of infection (Manca et al. 2018). Other miRNAs, including miR-148a and let-7a, contribute to immune modulation and cellular differentiation. Additionally, mEVs have been shown to be preferentially taken up by macrophages, thereby directly influencing immune cell activity (Garley et al. 2024).

**The diagnostic potential of milk-derived EVs** has been demonstrated through the identification of miRNAs like bta-miR-142-5p and bta-miR-223 as robust early biomarkers for mastitis, particularly in infections caused by Staphylococcus aureus (Saenz-de-Juano et al. 2022). Continuous multipoint sampling of mEVs has has confirmed the stability and clinical relevance of mEV miRNAs, such as bta-miR-223-3p, during subclinical mastitis (Saenz-de-Juano et al. 2022). The consistency of EV size and concentration under physiological stress further supports mEVs as reliable noninvasive diagnostic tools (Van Niel et al. 2018).

**Complementing milk EV analyses**, EVs isolated from peripheral blood and urine have emerged as promising systemic biomarkers for mastitis. Peripheral blood EVs demonstrate dynamic miRNA expression changes including upregulation of miR-21, miR-19a, miR-19b, and miR-320a, which correlate with disease progression and may serve as temporal indicators of infection severity (Ong et al. 2021; Luoreng et al. 2021). Urine-derived EVs provide additional diagnostic insights through metabolomic profiling that identifies metabolites associated with subclinical mastitis (Zwierzchowski et al. 2024). These multi-fluid biomarker approaches improve diagnostic sensitivity and reflect both local and systemic disease dynamics.

### EVs as potential platforms for precision drug delivery in mastitis

EVs, particularly mEVs, have been recognized as promising nanocarriers for targeted drug delivery in mastitis (Yong et al. 2022). These naturally occurring, biocompatible vesicles can encapsulate a diverse range of therapeutic agents, such as nucleic acids, lipids, proteins, and small molecules (Moghassemi et al. 2024). The immunoregulatory and antimicrobial features of these bacteria make them preferred candidates for treating mastitis, a common inflammatory condition affecting dairy animals (Saenz-de-Juano et al. 2022). By delivering anti-inflammatory agents directly to inflamed mammary tissue, EVs can mitigate excessive immune responses and enhance tissue repair (Xiong et al. 2024c). Furthermore, their ability to transport antimicrobial peptides or enhance immune cell function offers a dual therapeutic approach to combat bacterial infections (Wang et al. 2019). The natural stability and bioavailability of mEVs make them suitable for mouth-based or localized administration (Xia et al. 2024).

To enhance targeted delivery, researchers are engineering EVs with surface markers that specifically bind to receptors that are overexpressed in infected mammary tissue (Quinn et al. 2021). This strategy maximizes the therapeutic efficiency while minimizing systemic side effects. Furthermore, loading EVs with RNA-based therapeutics, such as microRNAs that regulate inflammatory pathways, shows promise in regulating host immune responses during mastitis (Du et al. 2021). Despite these advancements, challenges remain in the field of EV-based therapies, including efficient isolation techniques, scalability, and regulatory hurdles. Overcoming these obstacles is critical to realizing the full clinical potential of EVs for treating mastitis (Zhang et al. 2025).

### Role of EVs in physiological and pathological processes as biomarkers (fertility, embryo quality, placental quality, early abortion)

**Fig. 3:**
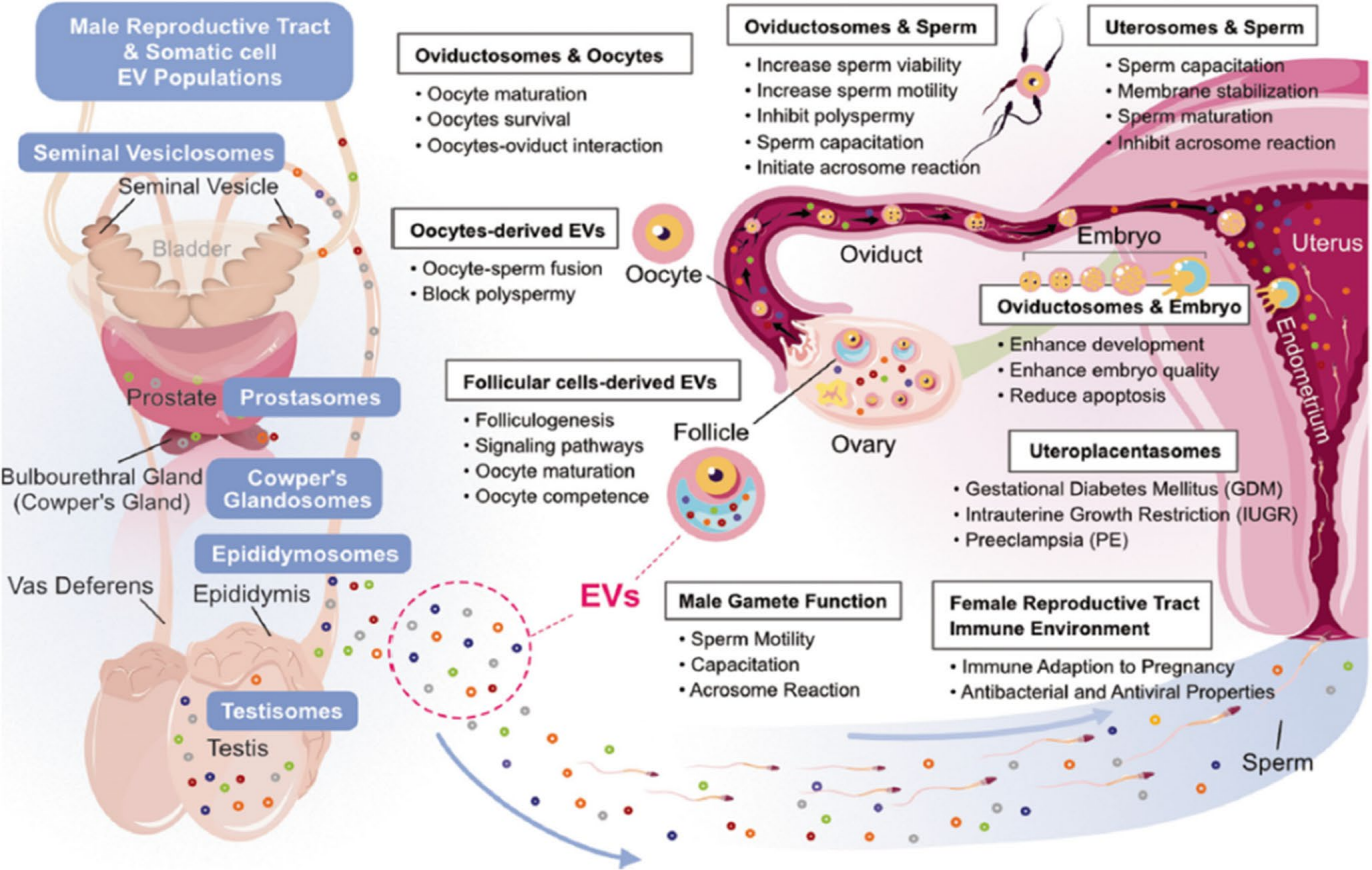
Schematic summarizes the diverse biological functions of EVs throughout the male and female reproductive tracts. In males, EVs derived from sources such as the testis, epididymis, prostate, and seminal vesicles contribute to sperm maturation, capacitation, motility enhancement, acrosome reaction initiation, and prevention of polyspermy. In females, EVs from follicular cells, oocytes, oviductal and uterine tissues facilitate folliculogenesis, oocyte competence, fertilization, embryo development, and modulate uterine immune environment to support pregnancy. (Republished from (Gurunathan et al. 2022)

Reproduction is a highly coordinated process that involves cellular growth and development via precise cell-to-cell communication among gametes, embryos, and the maternal environment (Godakumara et al. 2022). EVs are considered messengers that play a vital role in reproductive biology through the transfer of certain cargo (biological molecules), either in male or female genital tracts, whereas EVs are responsible for modulating transcription as well as translational processes such as normal follicular growth, oocyte maturation, proliferation and differentiation of granulosa cells, the fertilization rate, blastocyst formation, embryo development and implantation (Gurunathan et al. 2022) (Fig. 3).

### EVs as biomarkers for both the physiological and pathological processes of embryo and placental quality

Cell-to-cell communication is fundamental to physiological processes such as gametogenesis, fertilization, and embryonic development, which rely on intricate interactions across organ systems and tissues (Thompson et al. 2016) In multicellular organisms, this communication occurs via direct membrane contact, signaling molecules, chemokines, and notably, EVs (Théry et al. 2018). EVs have emerged as dynamic mediators of intercellular signaling, playing pivotal roles in both physiological and pathological contexts, particularly in reproduction and pregnancy (Capra and Lange-Consiglio 2020). These vesicles regulate signaling pathways critical for maternal-embryo harmony, ensuring successful fertilization, implantation, and pregnancy maintenance (Théry et al. 2018).

As forementioned, the EVs molecular cargo encompassing proteins, nucleic acids, and lipids—reflects the donor cell’s state, offering insights into tissue health and disease (Urabe et al. 2019). This specificity positions EVs as valuable biomarkers for diagnosing and monitoring reproductive health, including conditions like endometriosis, polycystic ovary syndrome (PCOS), and preterm birth (Capra and Lange-Consiglio 2020). EVs facilitate embryo-maternal crosstalk, modulating immune tolerance, placental development, and fetal growth. For instance, placental EVs contribute to immune modulation, preventing fetal rejection (Mincheva-Nilsson and Baranov 2014). while amniotic fluid EVs reflect gestational health, aiding in diagnosing preterm birth complications (Qamar et al. 2020).

In physiological contexts, EVs contribute to normal reproductive processes by promoting cellular cross-talk and creating an optimal environment for embryo development (Ng et al. 2013). Bovine oviductal EVs, for example, enhance the quality and developmental potential of in vitro-produced embryos (Lopera-Vasquez et al. 2017). These vesicles are hormonally regulated, with oviductal EV composition varying across the estrous cycle to support embryo quality (Capra and Lange-Consiglio 2020).

Embryonic EVs transport immunomodulatory factors like progesterone-induced-blocking factor 1 (PIBF), which sustains pregnancy by enhancing IL-10 production in maternal lymphocytes (Szekeres-Bartho et al. 2018). Furthermore, EVs carry pregnancy-specific miRNAs like miR-195-5p, which show significantly higher expression during the implantation window (day 19 post-AI) in buffaloes. The elevated EV-derived miR-195-5p modulates endometrial cell function by regulating key signaling pathways (PI3K-Akt, MAPK) and suppressing target genes, highlighting its potential as a diagnostic biomarker for early pregnancy detection (Kong et al. 2023).

Under pathological conditions, such as gestational diabetes mellitus (GDM) and preeclampsia, EV profiles are markedly altered. Placental EVs in preeclampsia exhibit pro-inflammatory, anti-angiogenic, and pro-coagulant effects, disrupting maternal vascular function (Chang et al. 2018). Similarly, hypoxia-induced EVs from trophoblasts modulate maternal immune responses, highlighting their diagnostic potential (Salomon et al. 2013). EVs isolated from bodily fluids—including blood, amniotic fluid, and uterine secretions—provide insights into the molecular dialog between the embryo and maternal tissues, reflecting both health and pathology (Qamar et al. 2020).

Recent discoveries, such as blebbisomes—large, motile EVs containing functional mitochondria—expand EV diversity, offering novel insights into their biomarker potential. These exceptionally large EVs (up to 20 μm in diameter) exhibit contractile behavior and carry active mitochondria, distinguishing them from other EVs and underscoring their role in intercellular communication (Tinnirello 2025).

In summary, EVs are indispensable in decoding embryo and placental health, bridging physiological adaptation and pathological disruption. Their specificity, stability in biofluids, and dynamic cargo position them as transformative biomarkers for optimizing maternal-fetal outcomes. By serving as carriers of information between cells, they unlock opportunities for diagnosing reproductive pathologies and developing targeted therapies, ultimately enhancing fertility and pregnancy success (Capra and Lange-Consiglio 2020).

### Biomarkers for embryo quality

In both human and veterinary medicine, in vitro embryo production (IVP) plays a pivotal role in enhancing pregnancy success. However, assessing embryo quality remains challenging due to limitations in traditional biomarkers. While genomic DNA was first detected in blastocoel fluid and embryo culture medium by Palini et al. (2013), mitochondrial DNA is unreliable for quality assessment because of contamination from maternal cumulus cells (Hammond et al. 2017). EVs, which are secreted by embryos into culture media and uterine environments, have emerged as promising biomarkers, reflecting embryo health through their cargo of miRNAs, DNA, and bioactive molecules (Galliano and Pellicer 2014).

Sexually dimorphic responses to environmental stressors, such as oxidative stress, are reflected in EV cargo: male bovine embryos exposed to high oxygen levels release more EVs enriched with transcription factors such as NFE2L2 and NOTCH1, highlighting EVs as sex-specific biomarkers of embryonic stress adaptation (Taqi et al. 2019).

EV release is correlated with embryonic physiological status. For example, under low oxygen tension, EV concentrations in culture media peak on Day 3 and decline by Day 7, with miR-210 identified as a hypoxia-sensitive biomarker (Andrade et al. 2019). Flow cytometry using propidium iodide staining further links EV quantity to embryo competence: fewer DNA-stained vesicles indicate reduced cellular damage and greater developmental potential (Pallinger et al. 2017). The differential expression and extracellular release of transcription factors, such as KLF4 and E2F1, into culture media further underscore the role of EVs in mediating sex-specific responses to oxidative stress, offering insights into embryo quality assessment (Taqi et al. 2019).

EVs isolated from individually cultured bovine embryo media are correlated with EV characteristics and developmental outcomes, with higher concentrations and smaller diameters observed in media from degenerating embryos than in those from blastocysts (Dlamini et al. 2025). These findings suggest that EV profiling, including concentration and size analysis, is a noninvasive biomarker for assessing preimplantation embryo quality and predicting developmental potential (Dlamini et al. 2025). However, miRNA profiling of culture media is complex. Elevated miRNA levels in degenerated bovine blastocysts may stem from passive leakage during cellular stress rather than active EV secretion, complicating their interpretation (Diamanti-Kandarakis et al. 2009).

EVs also influence oocyte maturation and embryo quality. Distinct miRNA profiles in follicular fluid EVs are associated with oocytes that yield high-quality embryos, highlighting their predictive value (Martinez et al. 2018). Compared with individually cultured embryos, group-cultured embryos exhibit superior developmental rates, suggesting that EVs mediate interembryonic communication by releasing survival-enhancing factors (Wydooghe et al. 2017). Species-specific studies in bovine, porcine, and human models have confirmed that embryo-secreted miRNAs are correlated with developmental robustness, suggesting that EVs are universal biomarkers (Dlamini et al. 2025).

Innovative studies underscore the therapeutic potential of EVs. Parthenogenetic embryo-derived EVs, enriched in pluripotency gene mRNAs, enhance cloned embryo development (Saadeldin et al. 2014). Coculture with EVs from human endometrial mesenchymal stem cells (EV-endMSCs) increases murine blastocyst cell numbers and growth factor secretion (e.g., VEGF and PDGF), improving viability (Marinaro et al. 2019). Similarly, uterine luminal fluid EVs (ULF-EVs) transfer miR-21 to embryos, increasing blastocyst rates and reducing the effects of apoptosis blocked by endocytosis inhibitors (Dlamini et al. 2025).

These findings suggest that engineered EVs carrying targeted miRNAs can be used to optimize in vitro embryo development. By harnessing EV-mediated communication, researchers can refine embryo selection criteria and culture protocols, advancing reproductive technologies in both clinical and agricultural settings.

### Extracellular vesicles as biomarkers for placental quality

The identification of reliable biomarkers for placental health is critical for the early detection of pregnancy-related pathologies, such as preeclampsia and gestational diabetes, which often remain asymptomatic until complications arise (Mitchell et al. 2015). EVs, particularly those of placental origin, have emerged as promising candidates because of their dynamic roles in feto-maternal communication and their ability to reflect placental functional status (Tannetta et al. 2014). Unlike other physiological conditions, pregnancy provides a temporally defined window for studying EVs, with placental tissue available postdelivery for comparative analysis, enabling the differentiation of placental EVs from those of maternal or fetal origin (Tannetta et al. 2014). Placenta-derived EVs are distinguishable via unique markers, including placental alkaline phosphatase (PLAP) and chromosome 19 miRNA clusters, which are exclusively expressed in placental tissue (Pan et al. 2025).

During gestation, placental EVs are secreted into the maternal circulation, where they modulate maternal immune tolerance through immunosuppressive signals, such as Fas ligand and HLA-G5, to prevent rejection of the semiallogeneic fetus (Capra and Lange-Consiglio 2020). These vesicles also promote a type 2 immune bias, which is essential for maintaining fetal antigen tolerance (Southcombe et al. 2011). Pathological conditions disrupt placental EV profiles: in preeclampsia, syncytiotrophoblast-derived EVs exhibit reduced levels of syncytin-2, a protein critical for cell fusion, whereas altered miRNA cargo (e.g., miR-210) is correlated with placental hypoxia and oxidative stress (Li et al. 2020). Similarly, gestational diabetes mellitus (GDM) alters the protein content of EVs, including miRNAs linked to insulin resistance, reflecting placental metabolic dysfunction (Jayabalan et al. 2019).

Environmental stressors, such as hypoxia and hyperglycemia, further influence EV biogenesis and bioactivity. Trophoblasts under low oxygen tension release EVs with elevated proangiogenic and immunomodulatory factors, which may exacerbate placental insufficiency in preeclampsia (Salomon et al. 2015). In domestic animals, where placentation is less invasive (e.g., epitheliochorial in ruminants), EVs may serve as critical biomarkers because of their ability to traverse placental barriers, offering insights into fetal health despite limited protein exchange (Qamar et al. 2020).

The diagnostic potential of placental EVs extends to their miRNA cargo, which regulates mRNA and protein expression in target cells, influencing implantation and fetal development (Capra and Lange-Consiglio 2020). For example, first-trimester placental EVs carry immunoregulatory miRNAs that shape the maternal uterine environment, ensuring successful implantation (Herrera-Van Oostdam et al. 2019). However, interspecies variations in placental structure necessitate further research to clarify EV trafficking mechanisms in nonhuman models (Qamar et al. 2020).

### Extracellular vesicles as biomarkers for fertility

EVs have emerged as pivotal mediators of reproductive health, offering noninvasive biomarkers for assessing fertility in both males and females. In females, EVs derived from oviduct epithelial cells and follicular fluid play critical roles in embryo development and cryotolerance. For instance, bovine oviduct EVs enhance blastocyst re-expansion and hatching postthaw by upregulating genes involved in trophectoderm integrity (e.g., aquaporins, Na+/K + ATPase) and tight junction assembly, thereby improving embryo resilience (Sidrat et al. 2022). Similarly, follicular fluid EVs isolated via size-exclusion chromatography (Yılmaz et al. 2023) enhance embryonic quality markers (*IFNT1*, *SOX2*) and increase blastocyst cell counts, underscoring the impact of isolation methods on EV functionality (Pérez-García et al. 2025).

In uterine health, EVs from mares with endometritis exhibit aberrant expression patterns linked to inflammation and apoptosis (Ibrahim et al. 2022), with HTRA1 identified as a potential biomarker for subclinical endometritis, in cows (Piibor et al. 2024). Circulating EVs in maternal blood also carry pregnancy-specific miRNAs (e.g., miR-25, miR-3596), which distinguish viable pregnancies from embryonic loss, offering promise for early pregnancy diagnostics (Pohler et al. 2017).

Studies have shown that EVs influence female reproductive processes, including maternal-fetal communication and follicular development (Roca et al. 2022). However, there is a relative shortage of research focusing on EVs as biomarkers for female fertility, particularly compared to studies on male fertility or other areas of reproductive health.

In males, seminal plasma EVs (SP-EVs) are rich in biomolecules that reflect semen quality and fertility. Recent studies in rabbits demonstrate that SP-EVs from fertile and subfertile males exhibit distinct miRNA profiles, with differential expression of 18 miRNAs (e.g., miR-190b-5p, let-7b-3p upregulated in fertile; miR-449a-5p, miR-146a-5p downregulated in fertile). These miRNAs are implicated in spermatogenesis, homeostasis, and infertility, suggesting their utility as biomarkers for male fertility (Sakr et al. 2023).

Proteins such as aquaporin-7 (AQP7) and TEX101 in SP-EVs further correlate with sperm motility and fertilization capacity, with reduced levels observed in subfertile individuals (Yu et al. 2023). Similarly, miRNAs like miR-34c-5p and miR-449a regulate pathways critical for spermatogenesis (e.g., MAPK, Wnt signaling), with dysregulation linked to abnormal semen parameters (Luo et al. 2024).

EVs also modulate sperm function directly; for example, boar SP-EVs inhibit premature acrosome reactions via the protein EZRIN, which regulates sperm-oviduct interactions (Xu et al. 2024). Stress-induced alterations in EV cargo, such as downregulation of miR-126-5p and miR-23b-5p in heat-stressed bulls, highlight their sensitivity to environmental perturbations (Alves et al. 2021). Additionally, EVs serve as predictors of semen cryotolerance, with specific miRNAs (e.g., ssc-miR-130a) in boar SP-EVs distinguishing freezable from low-freezability semen (Liu et al. 2020). Studies have demonstrated that EVs can modulate sperm functions and improve semen preservation, crucial for maintaining viability during storage (Roca et al. 2022).

The compositional differences in EVs between fertile and subfertile populations underscore their potential as noninvasive diagnostic tools (Rana et al. 2024). Furthermore, EVs may protect spermatozoa from oxidative stress and enhance assisted reproductive technologies (Tesfaye et al. 2021). However, further research is needed to standardize isolation methods, characterize EV subtypes, and confirm their functional roles in reproduction (Roca et al. 2022). Collectively, these findings underscore SP-EVs as noninvasive biomarkers for diagnosing male fertility and guiding assisted reproductive strategies (Table 1).

**Table 1:** Reproductive disorders: comparative analysis of EV-Based vs. Conventional therapies

Aspect	Traditional Treatments	EV-Based Therapeutics
Efficacy	Often involves hormonal therapies, surgery, or pharmaceuticals that manage symptoms but may not fully restore reproductive function. Variable success rates depending on disorder (Capra 2020).	EVs deliver bioactive molecules (miRNAs, proteins) targeting pathological pathways to promote tissue regeneration, immune modulation, and improved embryo development (Saadeldin et al. 2014) Early studies show promising effects on follicular growth and implantation.
Safety	Potential side effects include hormone imbalance, immune reactions, or surgical complications. Systemic treatments can cause off-target effects (Stawarska et al. 2024).	EVs are biocompatible, lack replicative potential, and have lower immunogenicity, reducing risks of adverse effects (Gnopo et al. 2017). Localized delivery reduces systemic toxicity.
Cost & Practicality	Conventional therapies are well-established and generally more affordable with existing infrastructure. May require repeated interventions (Stawarska et al. 2024).	Currently, EV isolation/production is costly and complex, but advances in scalable biomanufacturing are reducing these barriers (Kowal et al. 2014; Zhang et al. 2024). Potential cost savings via improved outcomes and reduced side effects.
Mechanism of Action	Broad systemic or hormonal intervention affecting entire physiological systems. Often symptom management (Wang et al. 2025a).	Targeted delivery of bioactive cargo (miRNAs, proteins) that modulate specific reproductive pathways, enabling more precise regulation of cellular processes (Stawarska et al. 2024).
Clinical Status	Established standard of care for many reproductive disorders worldwide (Stawarska et al. 2024).	Mostly preclinical and early clinical stages, with growing research but limited approved therapies (Dogrammatzis et al. 2020).

### Emerging roles of extracellular vesicles in equine physiology and sport horse performance

Recent veterinary research has begun to reveal the important roles that extracellular vesicles play in equine physiology, particularly in sport horses. According to research, EVs derived from synovial fluid in horses with osteoarthritis or tendon injury carry disease-specific protein cargos and may mediate some therapeutic effects of mesenchymal stem cell and platelet-rich plasma treatments, providing promising cell-free therapeutic alternatives (Kong et al. 2023). Furthermore, EV isolation from horse bronchoalveolar lavage fluid has allowed for extensive proteome characterisation, enhancing the model’s use in respiratory disease research and emphasizing the need of isolation procedures for EV purity (Kang et al. 2025).

Mesenchymal stem cell-derived EVs from equine adipose tissue have shown immunomodulatory effects in in vitro models of tendon injury and endometritis, both frequent illnesses in sport horses, highlighting their regenerative potential (Lanci et al. 2024). Furthermore, circulating EV concentrations and molecular profiles in horses change dynamically to physical activity, making them potential biomarkers and regulators of exercise adaptation and performance optimization (Lanci et al. 2024). These findings contribute to a better understanding of EVs as multifunctional modulators in equine health and sports performance.

### Extracellular vesicles in canine oncology: immunomodulation, cytokine dynamics, and diagnostic applications

Beyond their activities in general physiology, new research suggests that extracellular vesicles have an important role in canine oncology, notably in terms of immunomodulation and diagnostic value (Stawarska et al. 2024). EVs produced from canine B-cell lymphoma and leukemia cells have been shown to actively affect T and B lymphocyte activities, impacting cytokine secretion profiles and immune checkpoint expression in the tumor microenvironment (Kuang et al. 2025). For example, M1-polarized macrophage-derived EVs have been demonstrated to boost pro-inflammatory cytokines while decreasing immunosuppressive markers, implying their potential as immunomodulators in canine malignancies (Wang et al. 2024). Furthermore, the uptake of platelet-generated EVs by immune cells in dogs has a major impact on cytokine production, with higher levels of platelet-, leukocyte-, and T-cell produced EVs associated with enhanced pro-inflammatory states in dogs with various malignancies (Lanci et al. 2024).

This dynamic interplay demonstrates how EVs influence the immunological landscape during cancer progression (Yong et al. 2022). As a result, the significant differences in EV concentrations and molecular cargo profiles reported in canine malignancies suggest a promising avenue for diagnostic profiling in veterinary practice (Urabe et al. 2019). The quantification and analysis of EVs from various cellular origins provide a novel, non-invasive biomarker technique that has the potential to improve early cancer detection, illness monitoring, and therapy response assessment in clinical veterinary conditions (Kuang et al. 2025).

### Extracellular vesicles: dual roles in disease prevention and therapy

#### Preventive EVs: vaccine development and immunomodulation

EVs have emerged as promising tools in the fields of therapeutics and vaccine development because of their natural biocompatibility, ability to transport biomolecules, and capacity to stimulate immune responses (Schorey et al. 2015; Cheng and Hill 2022). Unlike traditional vaccine platforms that rely on live-attenuated or inactivated pathogens, EV-based vaccines offer a high safety profile with a reduced risk of reversion to virulence (Gnopo et al. 2017; Jorge and Dellagostin 2017; Xiong et al. 2024a).

The ability of EVs to mimic pathogen‒host interactions while avoiding the risks associated with live-attenuated or inactivated vaccines positions EVs as a transformative strategy for combating infectious diseases in livestock, poultry, and companion animals. Furthermore, EVs can be engineered to carry specific antigens or even nucleic acids, enabling targeted vaccine design that addresses the limitations of conventional approaches (Hao et al. 2021; El Safadi et al. 2024).

#### Biological benefits of EVs in vaccines

EVs, particularly exosomes, offer significant advantages over traditional veterinary vaccines (inactivated pathogens, subunit proteins, and viral vectors), which often suffer from limitations such as incomplete protection, safety concerns, and logistical challenges. EV-based vaccines address these issues through several key mechanisms.

1. Enhanced Antigen Presentation
  First, EVs enhance antigen presentation by containing adhesion molecules that facilitate uptake by antigen-presenting cells (APCs), improving immune system activation (Schorey et al. 2015). This enhanced presentation, along with the preservation of native antigen conformation on EVs (shown to elicit stronger immune responses than soluble proteins, particularly membrane-bound antigens in murine models (Hu et al. 2022), promotes robust humoral and cellular immunity.
2. Safety Advantages
  Second, EVs present inherent safety benefits compared to live or attenuated vaccines, as they lack replicative potential. This feature eliminates risks of reversion to virulence and reduces unintended immune reactions (Gnopo et al. 2017), making EV-based vaccines safer for veterinary use.
3. Stability and Biodistribution
  Third, EVs exhibit superior stability and improved biodistribution. Unlike conventional vaccines, EV-based vaccines maintain structural stability and are noninfectious, often demonstrating remarkable thermostability; lyophilized EVs can retain functionality at elevated temperatures (25–40 °C), bypassing the ultracold storage requirements of some newer vaccine technologies (Charoenviriyakul et al. 2018; Zhang et al. 2024). Their ability to circulate systemically and reach distal organs allows for more effective antigen dissemination and enhanced immune system interactions (Montaner-Tarbes et al. 2019). This improved biodistribution, coupled with cross-priming ability (the delivery of antigens to dendritic cells and macrophages for enhanced cellular and humoral responses (Gnopo et al. 2017), makes EVs particularly attractive.
4. Scalable Production
  Fourth, EV production can leverage existing biomanufacturing frameworks from cell therapy, enabling cost-effective large-scale production (Zhang et al. 2024).
5. Immune Modulation and Therapeutic Versatility
  Finally, the potential for immune modulation through the carriage of microRNAs and other molecules that influence immune responses makes EVs valuable for both prophylactic and therapeutic interventions. In essence, EVs mimic natural infection processes and interact with the immune system in a targeted and controlled manner, making them promising platforms for next-generation vaccine development (Tan et al. 2024).

In summary, The biologically inherent properties of EVs—such as enhanced antigen presentation, safety, thermostability, tissue targeting, scalable production, and immune modulation—highlight their immense potential as innovative veterinary therapeutic agents and vaccines. Organizing these advantages into thematic subsections clarifies their multi-faceted roles and facilitates reader comprehension.

### Advances in EV-based vaccines across pathogen types

#### Viral pathogens

Research into the use of EVs for immunization against viral diseases in livestock and companion animals has shown significant promise. One notable example is their application against porcine reproductive and respiratory syndrome virus (PRRSV), which is a major challenge in the swine industry (Montaner-Tarbes et al. 2018).

The serum-derived EVs from PRRSV-recovered pigs elicited robust virus-specific IgG and IFN-γ responses in vaccinated animals, resulting in protective immunity without adverse effects or viral replication (Montaner-Tarbes et al. 2018). Importantly, this EV-based strategy enables the differentiation between infected and vaccinated populations—a critical feature for managing outbreaks and facilitating surveillance. Further innovation was shown by engineering EVs to deliver microRNAs that target PRRSV receptors (sialoadhesin and CD163). This approach suppressed receptor expression in *Sus scrofa* cells and reduced viral titers, highlighting the dual utility of EVs as prophylactic and therapeutic tools. By interfering with the early stages of viral entry and replication, this method offers long-lasting antiviral protection (Kornicka-Garbowska et al. 2019; Montaner-Tarbes et al. 2019).

Similarly, advancements in combating foot and mouth disease virus (FMDV) have revealed the potential of antigen-presenting cell-derived EVs pulsed with FMDV antigens. These EVs stimulate B-cell activation, including the marginal zone and follicular subsets, by presenting native viral epitopes (Menay et al. 2024). This approach underscores the capacity of EVs to mimic natural antigen presentation, effectively priming adaptive immunity. In influenza research, murine models immunized with EVs expressing influenza nucleoproteins exhibited potent cytotoxic CD8 + T-cell responses, suggesting a scalable strategy to mitigate influenza outbreaks in livestock (Anticoli et al. 2018).

Collectively, these studies position EVs as transformative solutions for veterinary viral immunization, combining safety, targeted antigen delivery, and compatibility with DIVA (Differentiating Infected from Vaccinated Animals) principles—key advantages for controlling economically devastating viral diseases.

Despite these promising findings, research using EVs in veterinary viral diseases remains less common than studies involving other pathogens, such as parasites and bacteria. This disparity arises because viral replication inside host cells shares pathways with EV biogenesis, leading to potential confounding results. For example, during acute infection, both EVs and viruses are present in the host, complicating separation due to their similar sizes and densities (Chahar et al. 2018; Reyes-Ruiz et al. 2019; Dogrammatzis et al. 2020). Overcoming these technical challenges will be essential for advancing the field and expanding the application of EV-based vaccines against viral diseases in veterinary medicine.

#### Bacterial pathogens

EVs have also been studied as vaccine candidates against bacterial diseases. Outer membrane vesicles (OMVs) derived from gram-negative bacteria have shown significant promise as vaccine candidates. These vesicles are rich in pathogen-associated molecular patterns (PAMPs), such as lipopolysaccharides (LPS), porins, and other conserved antigens, which enable interactions with APCs and enhance phagocytosis. For example, gene-edited OMVs derived from *Staphylococcus aureus* provided protection against multiple strains in murine sepsis models, highlighting their potential as a novel platform for combating multidrug-resistant bacterial infections (Wang et al. 2018).

Several OMV-based vaccine candidates have advanced to preclinical stages, targeting pathogens such as *Vibrio cholerae*, *Klebsiella pneumoniae*, and *Bordetella pertussis* (Schild et al. 2008; Fransen et al. 2010; Wu et al. 2020).

Genetic detoxification strategies, such as removing toxic components while preserving immunogenic epitopes, have been employed to optimize the safety and efficacy of these vaccines (Gnopo et al. 2017). Additionally, studies on *Burkholderia pseudomallei* (∆purM strain) OMVs demonstrated that these vesicles, when combined with heterologous peptides, induced robust antibody and B-cell responses compared with traditional adjuvants such as alum or CpG DNA (Prior et al. 2021). These findings underscore the dual role of OMVs as standalone vaccines and potent adjuvants to increase the immunogenicity of conventional vaccines.

#### Parasitic pathogens

Parasite-derived EVs, often termed exosome-like vesicles, serve as a conserved mechanism for intercellular communication, mediating both parasite–parasite interactions and parasite–host crosstalk (Twu and Johnson 2014). These vesicles often contain proteins, nucleic acids, and lipids that modulate host immune responses, contributing to disease pathogenesis or protection. For example, EVs secreted by *Schistosoma mansoni* adult worms harbor miRNAs and proteins involved in host–parasite interactions, offering insights into their potential as vaccine candidates against schistosomiasis (Samoil et al. 2018).

In poultry farming, coccidiosis caused by Eimeria tenella is a major economic concern. Chickens vaccinated with dendritic cell-derived EVs pulsed with *Eimeria tenella* antigens exhibited enhanced humoral (IgG, IgA) and cellular immune responses, reduced gut lesions, and improved weight gain (Del Cacho et al. 2011). Similarly, EVs derived from *Toxoplasma gondii*-infected antigen-presenting cells induce protective immune responses in animal models (Jung et al. 2020). Notably, alum-adjuvanted exosomes elicited stronger humoral and cellular immunity than traditional excretory-secretory antigens (ESAs), reducing tissue cyst burdens (Tawfeek et al. 2023).

Proteomic analysis of *Fasciola gigantica* EVs revealed immunomodulatory proteins capable of enhancing host immunity, suggesting their utility as vaccine candidates against fascioliasis (Sheng et al. 2023). In schistosomiasis, which affects over 280,000 individuals annually and lacks an effective vaccine, Schistosoma mansoni-derived EVs have shown promise. These vesicles contain miRNAs and proteins that mediate host–parasite interactions, potentially serving as vaccine targets (Samoil et al. 2018; LoVerde 2019).

Furthermore, specific proteins and RNAs from different developmental stages of *Schistosoma japonicum* cercarial excretory-secretory products have been identified as potential vaccine targets, emphasizing the importance of EV-based approaches in controlling schistosome infections (Qadeer et al. 2021). EVs derived from *Plasmodium vivax*-infected individuals contain 23 novel parasitic proteins implicated in metabolic regulation, immune response induction, and T-cell activation (De Sousa et al. 2022).

Ticks, vectors for numerous zoonotic pathogens, pose significant challenges due to acaricide resistance and reservoir maintenance in wildlife. Recent studies evaluated EVs isolated from the salivary glands and midguts of *Amblyomma americanum* as vaccine candidates for white-tailed deer (*Odocoileus virginianus*).

Vaccination resulted in seroconversion and sustained increases in total IgG levels up to one year postvaccination, significantly increasing the on-host mortality of female ticks without adverse effects on nymphs or reproductive parameters (Gonzalez et al. 2025). Differential proteomic profiling of exosomes from high tick resistance (HTR) versus low tick resistance (Sakr et al. 2023). cattle breeds revealed distinct protein signatures associated with immunity/defense mechanisms (Turner et al. 2022). HTR cattle presented increased expression of proteins linked to B-cell activation, immunoglobulin production, and immune response modulation, whereas LTR cattle lacked such profiles. These findings emphasize the role of EVs in shaping host resistance to parasitic infections.

#### Challenges and future perspectives in EV vaccine development

The use of EVs in veterinary vaccine technology holds significant promise but also faces several challenges that must be addressed to facilitate their widespread adoption. A primary concern is the need for standardized methods for EV characterization, validation, and large-scale production (Kowal et al. 2014; Lener et al. 2015). Efficient production, purification, and characterization processes are essential to ensure consistency and cost effectiveness. Without such standardized protocols, translating EV vaccines from research to commercial application remains a formidable challenge.

Another challenge involves optimizing antigen loading onto EVs. Genetic engineering strategies can enhance antigen presentation on EV membranes, thereby increasing their immunogenic potential and making them more competitive with traditional vaccines (Prior et al. 2021). This could significantly improve the efficacy of EV vaccines, making them more competitive with traditional vaccines. Studies have demonstrated that optimizing cargo loading into EVs can significantly improve their therapeutic or immunological effects (Kim et al. 2016). However, further advancements in this area are necessary to fully realize the potential of EV-based vaccines.

Regulatory and safety considerations also pose significant barriers to the clinical translation of EV vaccines in veterinary medicine (Xiong et al. 2024b). Clinical trials evaluating the safety, stability, and immunogenicity of EV vaccines across different animal species are indispensable before commercialization can proceed (Vader et al. 2014; Lener et al. 2015). Quality control measures and adherence to regulatory guidelines are crucial for ensuring the reliability and safety of these therapeutics (Lener et al. 2015; Xiong et al. 2024b). These challenges highlight the need for further research and collaboration between academia, industry, and regulatory bodies to advance the field of EV-based vaccines in veterinary medicine.

A promising avenue for enhancing the efficacy of EV-based vaccines lies in combining them with adjuvants or advanced delivery systems. For example, hydrogels can enhance retention and immune stimulation, increasing the effectiveness of EV vaccines (Wang et al. 2019). Additionally, expanding research into emerging pathogens is vital. Given the growing threat of zoonotic diseases, EV-based vaccine strategies should be explored for novel pathogens that endanger both animal and human health.

Beyond vaccination, further research is needed to explore the full potential of EVs in veterinary applications, including their roles in regenerative and reproductive medicine (Zakirova et al. 2020). By addressing the current challenges and leveraging innovative approaches, the field of EV-based vaccines in veterinary medicine can advance significantly, offering transformative solutions to combat infectious diseases and promote animal health.

#### Therapeutic EVs: tissue regeneration and disease management

Extracellular vesicles have emerged as innovative therapeutic options in veterinary medicine because of their remarkable ability to modulate tissue repair, reduce inflammation, and regulate immune responses. In particular, EVs derived from mesenchymal stem cells (MSCs) have gained considerable attention because of their regenerative properties. Studies using animal models have shown that MSC-derived EVs can significantly enhance tissue repair and reduce inflammation, making them an attractive approach for treating musculoskeletal injuries, organ damage, and other conditions in animals (Liu et al. 2022). One of the most promising therapeutic applications of MSC-derived EVs is in promoting tissue regeneration. In particular, studies in large animal models, including horses, cattle, pigs, sheep, goats, and other livestock, have shown that EVs derived from MSCs can facilitate cartilage repair and stimulate the healing of injured tissues (Liu 2024).

For example, in horses, MSC-derived EVs have been used to support cartilage repair in joints following musculoskeletal injuries, and similar applications are being explored in other species, such as cattle and pigs. These results suggest that EVs could become a valuable treatment option to promote recovery and restore tissue function in large animal species (Bruno et al. 2009).

This highlights the potential of EVs as a less invasive and more efficient alternative to conventional treatments such as surgery or pharmaceutical drugs. In addition to their regenerative effects, MSC-derived EVs play a critical role in reducing inflammation, which is a key factor in the progression of various chronic diseases, including musculoskeletal and organ-related disorders (Liu 2024). For example, in bovine species, EVs isolated from milk have demonstrated potent anti-inflammatory effects and could be used as a therapeutic approach for conditions such as mastitis, an inflammatory disorder common in dairy cattle. These EVs can regulate immune responses, reduce the release of inflammatory cytokines, and promote tissue healing, making them promising solutions for managing inflammation-related diseases in livestock (Lanci et al. 2024).

In addition to their ability to treat musculoskeletal injuries and inflammation, EVs derived from MSCs have demonstrated promising therapeutic potential in treating various other conditions in animals. In models of organ injury, such as liver or kidney damage, these vesicles have been shown to reduce inflammation, promote tissue regeneration, and enhance organ function (Grange and Bussolati 2022). The therapeutic advantages of MSC-derived EVs are mainly their ability to transport a diverse array of bioactive molecules, such as growth factors, RNA, and proteins, which support tissue repair and immune regulation. This versatility underscores the wide range of applications for EVs in treating various diseases and animal injuries (Liu et al. 2022).

MSC-derived EVs also offer several advantages over traditional therapeutic approaches, including cell-based therapies and pharmaceuticals. One of the key benefits of EVs is their ability to evade immune rejection, making them safer for animal use. Additionally, EVs can carry a diverse array of therapeutic cargo, allowing for targeted delivery to specific tissues or cells. This targeted delivery enhances the precision and effectiveness of the treatment, providing a more efficient therapeutic approach for various veterinary applications (Lanci et al. 2024).

As research into the therapeutic potential of EVs has advanced, these vesicles are expected to become increasingly significant in the field of veterinary medicine (Deng et al. 2017). The ability to promote tissue regeneration, reduce inflammation, and modulate immune responses positions EVs as an exciting and innovative approach for treating a wide variety of conditions in animals. As more is understood about the properties and mechanisms of EVs, they are expected to become an integral part of therapeutic strategies for improving animal health and treating diseases across species (Su et al. 2024).

In summary, extracellular vesicles, especially those derived from mesenchymal stem cells, hold significant promise in veterinary applications. Their regenerative properties, anti-inflammatory effects, and ability to promote tissue repair make them valuable tools for treating a wide range of conditions in animals. With continued advancements in research, EV-based therapies are poised to become a cornerstone of veterinary medicine, providing a safer and more targeted approach to treating a variety of ailments in animals, from livestock to companion species.

Rabbits are increasingly recognized as valuable preclinical models in biomedical research, particularly for evaluating the therapeutic potential of extracellular vesicle-based treatments (Lener et al. 2015). Owing to their small size, rapid reproduction rates, and utility in modeling various human diseases, they are ideal candidates for studying the effects of novel therapies (Bruno et al. 2009). In recent years, EVs derived from mesenchymal stem cells (MSCs) have demonstrated remarkable regenerative capabilities in rabbit models, especially for conditions involving tissue injury and degeneration (Liu et al. 2022). The use of rabbits to study the therapeutic potential of MSC-derived EVs offers promising insights into their application in veterinary and human medicine. A significant area of research has focused on the role of MSC-derived EVs in promoting tissue repair and regeneration in rabbits, particularly for skin and tendon injuries (Bruno et al. 2009). Studies have shown that these vesicles accelerate wound healing by stimulating key cellular processes such as migration, proliferation, and angiogenesis (Su et al. 2024).

The proangiogenic proteins and microRNAs (miRNAs) of EVs are believed to play critical roles in these regenerative processes. These bioactive molecules enhance tissue remodeling and the formation of new blood vessels, which are essential for optimal tissue repair. These findings underscore the therapeutic potential of EVs as a less invasive and effective treatment for tissue damage in rabbits, offering an alternative to more conventional therapies such as surgery or topical treatments (Deng et al. 2017). In addition to their use in wound healing, MSC-derived EVs have shown promise in the treatment of osteoarthritis in rabbits, a condition commonly used in animal models to study degenerative joint diseases (Komuro et al. 2022). OA is characterized by the progressive breakdown of cartilage and chronic inflammation within joints. In rabbit models, MSC-derived EVs have been shown to reduce inflammation in affected joints and stimulate cartilage regeneration (Bruno et al. 2009).

The therapeutic properties of these EVs, particularly their ability to modulate immune responses and promote tissue regeneration, provide an innovative approach for treating OA. This is especially significant for rabbits, as it presents a less invasive option for managing arthritis than traditional methods such as joint surgery or long-term drug treatments. Moreover, these EV-based therapies have the potential to improve the overall quality of life for rabbits suffering from OA and could eventually be translated to applications in other animal species with similar joint conditions (Bruno et al. 2009). The ability of MSC-derived EVs to address both acute injuries and chronic degenerative diseases in rabbits highlights their broad therapeutic potential. In addition to their ability to treat wound healing and osteoarthritis, EVs are being investigated for their ability to treat a variety of other conditions, including organ damage, inflammation, and even neurological disorders (Liu et al. 2022).

The versatility of EVs, combined with their natural capacity to deliver a diverse array of therapeutic molecules, makes them powerful candidates for a wide range of veterinary applications. Moreover, the use of rabbits as preclinical models to explore the effects of EV-based therapies could have far-reaching implications for both animal and human health (Linares et al. 2015). As research continues to unravel the mechanisms underlying EV-mediated tissue repair and immune modulation, these vesicles may become a cornerstone of regenerative medicine. The promising results observed in rabbit models suggest that EVs could offer a safer, less invasive, and more efficient alternative to traditional treatments, not only for rabbits but also for other animals suffering from similar conditions (Liu et al. 2022).

In summary, the therapeutic potential of MSC-derived EVs in rabbits holds great promise for advancing veterinary medicine. The ability of EVs to enhance tissue repair, decrease inflammation, and regenerate cartilage in various animal models positions them as a revolutionary treatment option. As the field of EV-based therapy continues to grow, these vesicles will likely become an integral part of treatment strategies for both veterinary and human health, providing innovative solutions for a variety of diseases and injuries (Table 2).

**Table 2:** Comparative analysis of extracellular vesicle mechanisms and therapeutic outcomes across veterinary species and diseases

Species/Model	Disease/Condition	EV Source	Key Mechanisms/Targets	Therapeutic or Diagnostic Outcome	Key Ligands/Molecular Targets	Reference(s)
Horses (Sport horses)	Osteoarthritis, tendon injury	Synovial fluid, MSC-derived EVs	Modulation of inflammation, tissue repair, immunoregulation	Cartilage regeneration, tendon healing, less inflammation	TGF-β, IGF-1, CD73, integrins, miR-140	(O’Brien et al. 2023)
Dogs	B-cell lymphoma, leukemia	Tumor-derived EVs	T/B cell modulation, cytokine secretion, immune checkpoint regulation	Tumor microenvironment modulation, diagnostic markers	PD-L1, MHC-I/II, Foxp3, CTLA-4, CD40L	(Żmigrodzka et al. 2022)
Dogs	Cancer (various)	Platelet/leukocyte EVs	Cytokine release modulation, inflammatory markers	Diagnostic EV profiling, inflammation assessment	CD41, IL-6, TNF-α, CCL2, CD45, CD61	(Żmigrodzka et al. 2019)
Cattle	Mastitis	Milk-derived EVs	Immune regulation, anti-inflammatory signaling	Inflammation reduction, mammary tissue repair	Lactadherin, TGF-β, miR-223, annexin V	Lanci et al. 2024
Swine	Viral infections (PRRSV)	Serum-derived EVs	Antigen presentation, miRNA modulation of viral entry	Protective immunity, reduced viral replication	PRRSV N protein, miR-181, MHC molecules	Montaner-Tarbes et al. 2018; Kornicka-Garbowska et al. 2019
Rabbits	Wound healing, osteoarthritis	MSC-derived EVs	Angiogenesis, immune modulation, tissue regeneration	Enhanced repair, reduced joint inflammation	VEGF, FGF2, miR-21, CD81, TSG101	Bruno et al. 2009; Komuro et al. 2022

#### Limitations and future research directions

A notable technical limitation in extracellular vesicle (EV) research involves potential batch effects arising during EV isolation and downstream analyses. These batch effects may result from variability in sample handling, reagent lots, operator technique, or instrument performance, potentially influencing EV yield, purity, and molecular profiling. To mitigate such variability, we employed standardized protocols, included internal controls where feasible, randomized sample processing, and monitored data for batch-associated variation. Despite these efforts, residual batch effects cannot be entirely excluded and should be considered when interpreting results and comparing data across experiments.To overcome these challenges and advance EV applications in veterinary medicine, coordinated future research efforts are essential:

- Give priority to Multi-Institutional Clinical Trials in Companion Animals.
  Large-scale, multi-center clinical studies are needed to rigorously evaluate EV-based diagnostics and therapies across diverse natural diseases in dogs, cats, horses, and other veterinary species. These collaborative trials will validate safety, efficacy, and clinical outcomes, while promoting harmonized protocols and facilitating veterinary regulatory approvals.
- Standardize EV Isolation, Characterization, and Reporting Methodologies.
  Development and widespread adoption of species- and context-specific guidelines building upon international standards such as MISEV are critical to minimize batch effects and technical variability. Establishing harmonized methodologies covering sample collection, EV isolation techniques, characterization tools, and data reporting will enhance reproducibility and comparability across studies.
- Foster Interdisciplinary Collaboration for EV Engineering and Therapeutics.
  Partnerships between veterinary clinicians, molecular biologists, bioengineers, and material scientists will accelerate the design of engineered EVs with enhanced targeting, cargo delivery, stability, and controlled release. Such efforts can drive innovation in biomaterials, scalable biomanufacturing, and precision veterinary therapeutics.
- Advance Fundamental and Systems-Level Research on EV Biology in Veterinary Species.
  Deeper understanding of EV biogenesis, cargo selection, biodistribution, and intercellular signaling in diverse veterinary species is needed. Integration of omics technologies (transcriptomics, proteomics, lipidomics) and systems biology approaches will aid in identifying novel biomarkers and therapeutic targets, clarifying EV roles in health and disease, and addressing heterogeneity and subtype distinctions.

By addressing technical limitations such as batch effects through standardized procedures and by investing in collaborative, multidisciplinary research, the veterinary field will be well-positioned to translate EV technologies effectively into clinical practice. These advances will improve animal health outcomes, foster innovation in veterinary diagnostics and therapeutics, and provide valuable translational insights applicable to human medicine.

## Conclusion

Extracellular vesicles have a broad range of applications, from serving as disease biomarkers to offering promising alternatives in drug delivery and cancer therapy. Their stability, targeting capabilities, and ability to cross biological barriers make them a key focus in biomedical research. The use of extracellular vesicles has become a promising approach for therapeutic applications in veterinary medicine, particularly for treating injuries, inflammatory conditions, and degenerative diseases in animals and rabbits. Their ability to transfer bioactive molecules that promote tissue regeneration and modulate immune responses makes them an exciting alternative to traditional treatments. As research continues to uncover the mechanisms by which EVs mediate these effects, EV-based therapies are anticipated to play a key role in the future of both veterinary and human healthcare, offering safer, more effective, and potentially cross-species treatment options for a variety of diseases.

## Data Availability

No datasets were generated or analysed during the current study.
